# Assessment of Upper Limb Motor Dysfunction for Children with Cerebral Palsy Based on Muscle Synergy Analysis

**DOI:** 10.3389/fnhum.2017.00130

**Published:** 2017-03-23

**Authors:** Lu Tang, Xiang Chen, Shuai Cao, De Wu, Gang Zhao, Xu Zhang

**Affiliations:** ^1^Department of Electronic Science and Technology, University of Science and Technology of ChinaHefei, China; ^2^Department of Children's Neurorehabilitation, First Affiliated Hospital of Anhui Medical UniversityHefei, China

**Keywords:** cerebral palsy, electromyography, non-negative matrix factorization, muscle synergy analysis, upper limb assessment

## Abstract

Muscle synergies are considered to be building blocks underlying motor behaviors. The goal of this study is to explore an objective and effective method to assess the upper limb motor dysfunction of cerebral palsy (CP) children from the aspect of muscle synergy analysis. Fourteen CP children and 10 typically developed (TD) children were recruited to perform three similar upper limb motion tasks related to the movements of elbow and shoulder joints, and surface electromyographic (sEMG) signals were recorded from 10 upper arm and shoulder muscles involved in the defined tasks. Non-negative matrix factorization algorithm was used to extract muscle synergies and the corresponding activation patterns during three similar tasks. For each subject in TD group, four muscle synergies were extracted in each task. Whereas, fewer mature synergies were recruited in CP group, and many abnormal synergy structures specific to CP group appeared. In view of neuromuscular control strategy differences, three synergy-related parameters were proposed and synergy structure similarity coefficient was found to have high ability in depicting the inter-subject similarity within task and the intra-subject similarity between tasks. Seven upper limb assessment (*UPA*) metrics, which were defined as the combinations of synergy structure similarity coefficients of three tasks, were proposed to assess the upper limb motor function of CP children. The experimental results demonstrated that these *UPA* metrics were able to assess upper limb motor function comprehensively and effectively. The proposed assessment method can serve as a promising approach to quantify the abnormality of muscle synergies, thus offering potential to derive a physiologically based quantitative index for assessing upper limb motor function in CP clinical diagnosis and rehabilitation.

## Introduction

Cerebral palsy (CP) describes a heterogeneous group of disorders affecting the development of movement and posture. This illness is caused by non-progressive insult to the developing brain (Bax et al., 2005; Butler, 2011). Patients with CP often suffer from neurological and physical abnormalities (Rosenbaum et al., 2007). In clinical treatment, clinicians often establish personalized therapeutic schedule for CP patients according to the severity of their abnormalities. Therefore, accurate assessment of the motion dysfunction is very important. Nowadays, the Gross Motor Function Measure (GMFM; Russell et al., 1989) has been widely adopted to measure the gross motor function of the CP children in clinical, particularly the functional changes over time. Apart from measuring the whole body motor function, the assessment of body partial motor function was also developing gradually. Some assessment methods, such as the Melbourne Assessment of Unilateral Upper Limb Function (Johnson et al., 1994), the Shriners Hospital for Children Upper Extremity Evaluation (SHUEE; Davids et al., 2006), and Fugl-Meyer Assessment (FMA; Fugl-Meyer et al., 1975) scale were frequently used in the clinic for the upper limb function assessment of CP patients (Krebs et al., 2009). Above methods are usually based on subjective, observational analysis of the ability of the patient to perform numerous tasks. On the other hand, motion analysis on the basis of three-dimensional kinematics offered an objective method for motion function assessment. Researchers often used motion analysis to characterize joint kinematics and the duration, velocity, smoothness and trajectory of movement, which can provide important information regarding the quality of upper limb motion (Mackey et al., 2005; Petuskey et al., 2007; Kontaxis et al., 2009; Butler, 2011). Additionally, as CP patients often suffered from abnormal muscle function such as muscle weakness and myotonia, which resulted in the abnormal pattern of surface electromyographic (sEMG) signal, researchers made some achievements in assessing CP motion abnormalities taking use of such phenomenon. Most sEMG-related studies took abnormal gait assessment as research target (Bojanic et al., 2011; Van Gestel et al., 2012; Zwaan et al., 2012; Torricelli et al., 2014), and some gait parameters extracted from sEMG signals were considered effective for gait analysis (Bojanic et al., 2011; Van Gestel et al., 2012). For instance, the mean frequency of sEMG has a potential capability to evaluate the functional muscle strength during gait in CP children (Van Gestel et al., 2012).

In the past few years, the concept of muscle synergies has been used to study complex motor control patterns. Plenty of evidences in support of the view that the central nervous system (CNS) may generate motor commands through a linear combination of a set of muscle synergies have been presented (Saltiel et al., 2001, 2005; d'Avella et al., 2003; Bizzi et al., 2008), and decomposition techniques applied to EMG data recorded form related muscles have shown that muscle synergies underlying postural responses were limited (Loeb et al., 1999; Ting and Macpherson, 2005; Todorov et al., 2005; Isa et al., 2007; Bizzi et al., 2008; Drew et al., 2008; Lacquaniti et al., 2012). In animal studies, D'Avella and Bizzi observed that five functional muscle synergies extracted during walking, jumping, and swimming of frogs were similar. Three synergies of the five were shared across behaviors whereas others were behavior-specific (d'Avella and Bizzi, 2005). Similar results have been demonstrated in humans. Ivanenko found that five basic temporal activation components were likely to be controlled and shared in the voluntary motor tasks of walking, during which subjects kicked a ball, stepped over an obstacle, or reached down and grasped an object on the floor (Ivanenko et al., 2004, 2005). For upper limb movement, d'Avella et al. found that the muscle activity of upper limb during diverse movements can be characterized by a definite set of muscle synergies (d'Avella et al., 2006).

With the muscle synergy framework, a few studies have also done some research on the muscle coordination patterns of patients with neuromuscular diseases. These studies suggested that muscle synergy patterns should possibly be used as physiological markers of the condition of patients with trauma, to guide the development of different rehabilitation approaches (Bizzi et al., 2008; Routson et al., 2013; De Groote et al., 2014), and to explain the motor impairment of patients with neuromuscular diseases. Fewer muscle synergies or different synergy structures were found to account for muscle activation of lower (Clark et al., 2010) and upper (Roh et al., 2013) limb movements in stroke survivors compared with healthy controls. This abnormality was observed to be slightly recovered after treatment (Tropea et al., 2013). In the study of the affected arm of stroke survivors, the preservation of normal muscle synergies in subjects with mild-to-moderate impairment was revealed, while merging and fractionation were found in severely impaired subjects (Cheung et al., 2009, 2012). Researchers also revealed that children with cerebral palsy used a simpler neuromuscular control strategy during gait compared to unimpaired individuals. Specifically, CP children recruited fewer synergies during walking in contrast with healthy controls (Schwartz et al., 2014, 2016; Torricelli et al., 2014; Steele et al., 2015; Tang et al., 2015).

Considering muscle synergy abnormality can reflect the motor dysfunction and physiological changes of neuromuscular diseases, we believe that muscle synergy analysis has a great potential in the assessment of motor impairment. The goal of this study is to explore an objective and effective method to assess the upper limb motor dysfunction of cerebral palsy children from the aspect of muscle synergy analysis. For the upper limb motor function, CP patients often have difficulty with the timing and coordination of reaching movements (Steenbergen et al., 1998). Therefore, three upper limb reaching motion tasks were designed, and the muscle synergy structures and activation patterns extracted from these three tasks in CP children and typically developed (TD) children were analyzed and compared. Based on the differences of muscle synergy structures and activation patterns between CP and TD group, the feasibility of the quantitative evaluation of the upper limb motor dysfunction of CP children was explored.

## Materials and methods

### Subjects

Twenty-four subjects, including 10 typically developed children (TD group, three males and seven females, 8.9 ± 2.7 years) and 14 children with CP (CP group, nine males and five females, 8.2 ± 2.6 years), were involved in this study. All the subjects in TD group were right-hand dominant, with no known neurological diseases, no muscular or skeletal impairment history of the upper limbs and the trunks, and no motion functional abnormalities. The inclusion criteria for CP children included being diagnosed with cerebral palsy clinically and having ability to complete the experiments independently, and the exclusion criteria for CP children were: (1) individuals were diagnosed with severe concurrent medical problems; (2) individuals who had undergone surgical therapy; (3) individuals had cognitive impairment or affective dysfunction that affects the understanding of the task instructions. The tested side was chosen to be the side with poorer motor function. For CP subjects, the motor function of the tested arm was reported through the FMA by a clinician right before the experiment. FMA scale has 33 items of upper extremity (FMAu) with a total of 66 scores. Evidently, each healthy subject had a score of 66 by FMAu evaluation. The demographic information of CP children was listed in Table 1. All the children and their guardians were informed of the experiment procedure, and signed an informed consent approved by Ethics Review Committee of Anhui Medical University (No. PJ 2014-08-04).

**Table 1:** **Information of children with Cerebral Palsy**.

**Subjects**	**CP1**	**CP2**	**CP3**	**CP4**	**CP5**	**CP6**	**CP7**	**CP8**	**CP9**	**CP10**	**CP11**	**CP12**	**CP13**	**CP14**
Gender	F	F	M	F	F	M	F	M	M	M	M	M	M	M
Age	8	12	8	8	5	10	13	8	4	6	8	6	8	11
Side	R	R	R	R	R	L	L	L	R	R	R	L	R	R
Type	SP	A	A	SQ	SQ	SH	SQ	SQ	A	SP	A	SP	MIX	SQ
GMFCS	II	I	III	III	II	I	II	II	I	III	I	II	III	III
FMAu	60	57	43	52	40	56	54	43	59	38	44	61	31	28
Speed (cm/s): Task1	20	21	18	18	19	17	20	14	19	7	11	18	8	11
Speed (cm/s): Task2	23	20	17	19	21	20	15	16	21	13	15	14	10	12
Speed (cm/s): Task3	21	23	20	16	20	22	16	19	22	13	17	17	12	14

*Type, Type of CP; SP, Spastic paralysis of the lower limbs; A, Athetoid; SQ, Spastic Quadriplegia; SH, Spastic Hemiplegia; MIX, Athetoid and Ataxia. GMFCS, Gross Motor Function Classification System. High GMFCS scale means bad motor function. FMAu, Fugl-Meyer assessment of upper limb and the maximal score is 66. Low FMAu means bad motor function of upper limbs*.

### Three motion tasks

Upper limb movements are usually realized by the coordination of shoulder joint, elbow joint, wrist joint, and fingers. Three tasks which could comprehensively reflect the extension/flexion of elbow and the adduction/abduction of shoulder joints, were designed to assess the upper limb motor dysfunction from the aspect of gross motor.

**Figure 1:**
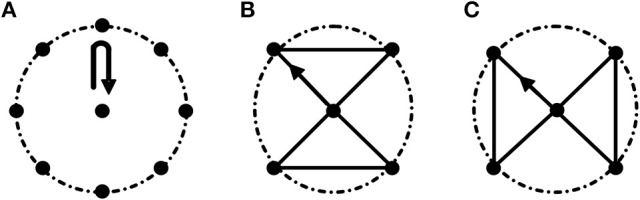
**Three motion tasks**. **(A)** Task 1. Center-out-center reaching task; **(B,C)** Path-movement tasks (Task 2 and Task 3, respectively). The radius of the dashed circle is 20 cm. All the tasks were started from the center point. In Task 2 and Task 3, subjects were asked to move cylinder along the black line.

Task 1: center-out-center reaching task. Subjects performed this task by moving a cylinder (Height: 8 cm; Radius: 1.5 cm) clockwise from the center point to eight equidistant points arranged along the circumference (Radius: 20 cm; Figure 1A. Movement route length of Task 1 is 320 cm.). This task mainly focuses on the extension and flexion of elbow joint accompanied with slight adduction and abduction of shoulder joint.

Task 2 and Task 3: path-movement tasks. Subjects performed path-movement tasks by moving cylinder along the direction of the arrow as shown in Figures 1B,C, respectively. Movement route length of Task 2 and Task 3 is 136.6 cm. These two path-movement tasks primarily involve the combination movements of elbow (extension/flexion) and shoulder (adduction/abduction) joints.

As shown in Figure 1, the reached points in Task 2 and Task 3 all were all included in Task1, and the routes of Task 2 and Task 3 were designed based on the follow criterions: (1) The length of the routes was the same; (2) The maximum range of activity in two path-movement tasks was the same. In all three tasks, subjects were asked to move cylinder along the fixed routes. To be clear, every line was formed by the grooves with width of 1.7 cm and depth of 3 cm, and the routes are connected with all the grooves. Before starting the experiment, subjects performed a simple learning process under the guidance of professionals. During the experiment, subjects seated upright in front of a height adjustable table and carried out the tasks in an inclined plane which has 45 degrees of angle between the plane and the desktop. During the experiment, the subject's wrist was tied with a 20 cm long, 10 cm wide strap to restrain wrist movements. Three 100 cm long, 5 cm wide straps were used to restrain trunk movements, one was used to tie the chest to the back of the chair in horizontal direction, and other two straps were fastened in vertical direction to keep the upper body upright. As CP subjects usually couldn't control the speed very well due to motor dysfunction, they were asked to complete the tasks at self-selected speed. With the known movement route length of a given task and the time to complete the task, the speeds of CP subjects were estimated and shown in Table 1. The velocity of TD subjects were 15 ± 1.7 cm/s. During the experiment, subjects were instructed to carry out a series of trials (15–20 times per task) and were encouraged to keep their speed consistently with the wrist and forearm not touching the table.

### Data acquisition

**Figure 2:**
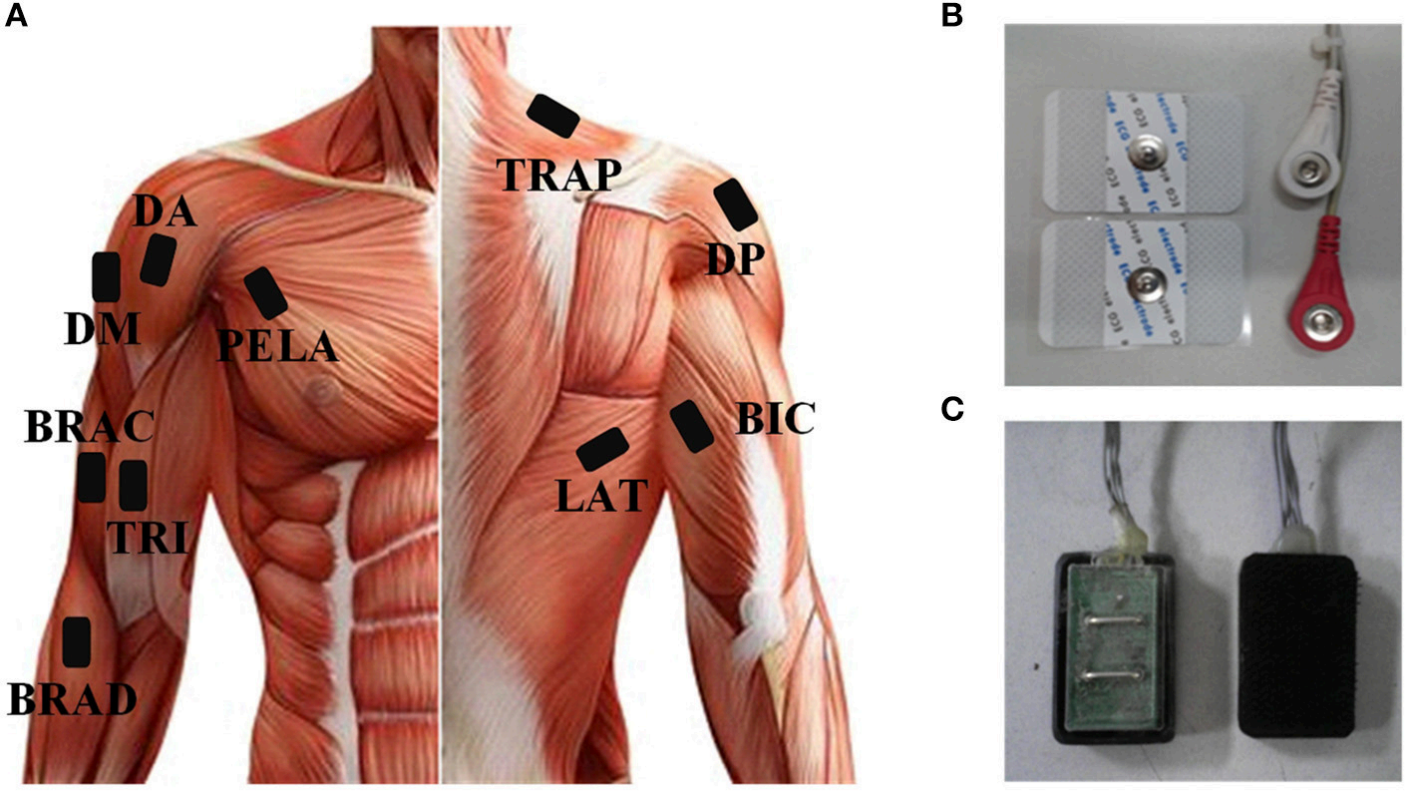
**(A)** The placement of sEMG and ACC sensors. **(B)** Disposable self-adhesive electrodes. **(C)** Bipolar Ag-AgCl surface electrodes.

As the subjects performed the tasks, sEMG signals were recorded from 10 upper arm and shoulder muscles (Figure 2A) including: brachioradialis (BRAD), brachialis (BRAC), biceps brachii (BIC), triceps brachii (TRI), anterior deltoid (AD), medial deltoid (MD), posterior deltoid (PD), latissimus dorsi (LAT), upper trapezius (TRAP), and pectoralis major (PECM). In order to get high quality of sEMG signals, three bipolar Ag-AgCl surface electrodes were placed on BRAD, BIC, and TRI. Four disposable self-adhesive electrodes were placed on the other muscles (Figures 2B,C). Electrodes were placed in accordance with the guidelines of surface EMG for non-invasive assessment of muscles (SENIAM; Hermens et al., 2000). The reference electrode was placed over the left electrically neutral lateral epicondyle (Tropea et al., 2013). Each recorded site was cleaned with alcohol before placing the electrodes. All the data were collected by a home-made 16-channel sEMG system (band pass filter: 20–500 Hz; A/D resolution: 24-bit; gain: 1,680 times) and the sampling rate was set to 1,000 Hz. Each trial had a data file in which data was recorded at the beginning of a trial and saved at the end of this trial. All data were recorded and stored to a laptop computer via USB for further analysis using a customized program in Matlab 7.1.4 (The Mathworks, Natick, MA).

### Muscle synergies extraction andanalysis

#### sEMG pre-processing

Before extracting muscle synergies, the collected sEMG signals were normalized to unit variance, in order to eliminate the amplitude difference resulted from electrode offset across trials and subjects. Then, the signals were pre-processed through high-pass-filtering (window-based finite impulse response filter, 50th order, cutoff at 40 Hz), rectification, and low-pass-filtering (window-based finite impulse response filter, 50th order, cutoff at 20 Hz; Cheung et al., 2009). Finally, the pre-processed sEMG signal from each muscle was normalized to its peak value.

#### Extraction of muscle synergies

The extraction of muscle synergies was based on the decomposition of the pre-processed sEMG signals (***V***_*m*×*t*_, *m* is the number of muscles). With non-negative matrix factorization algorithm (NMF; Lee and Seung, 1999), ***V***_*m*×*t*_ was decomposed into two matrices: ***W***_*m*×*t*_ and ***C***_*n*×*t*_, where ***W***_*m*×*t*_ is the muscle synergy matrix (*n* is the number of synergies, and the dimensions of each synergy vector were the same as the number of the recorded muscles) and ***C***_*n*×*t*_ is the synergy activation coefficient matrix. Muscle synergy matrix is functionally activated by a specific activation coefficient matrix (Clark et al., 2010), and the coefficient ***C***_*n*×*t*_ represents the neural command that specifies how much each synergy contribute to muscle's total activation. In the process of factorization, the matrices of ***W*** and ***C*** were initiated with random non-negative synergies and random coefficients, and then the NMF algorithm performed an iterative optimization until the variability accounted for (VAF) reached a threshold (Lee and Seung, 1999). Assuming that the reconstructed matrix ***Vr***_*m*×*t*_ could be expressed as Equation (1), the minimum number of muscle synergies were estimated according to the variability accounted for (VAF) shown in formula (2).

(1) $$\begin{array}{r} \text{V}{\text{r}}_{m\times t}={\text{W}}_{m\times n}\times {\text{C}}_{n\times t} \end{array}$$

(2) $$\begin{array}{r} \text{VAF}=1-{\left({\text{V}}_{m\times t}-\text{V}{\text{r}}_{m\times t}\right)}^{2}/{{\text{V}}_{m\times t}}^{2} \end{array}$$

Each subject did S (15 ~ 20) trials for each task. To ensure the extracted muscle synergies can adequately compose the original matrix ***V***_*m*×*t*_, the VAF values were estimated by gradually increasing the number (*n*) of the synergy (starting from one to the number of muscles). Moreover, to maximize the chance of using a VAF value corresponding to a global optimum in the NMF analysis, synergy extraction was repeated 5000 times with random initial estimates of the matrix ***W*** and ***C*** in each number (Roh et al., 2013). When the mean of the VAF was larger than 0.95 (Independent sample *T*-test, *p* < 0.05), the number of muscle synergies was determined and the muscle synergy extraction process was aborted.

The main goal of this study is to explore an objective method to effectively assess the upper limb motor dysfunction of cerebral palsy (CP) children in view of muscle synergy differences of three similar motion tasks between the control group and CP group. Therefore, reliable extraction of task-related muscle synergies is very important for the feasibility of the proposed method. In this study, muscle synergies were extracted from the data of individual trial from each task, and then averaged across trials. For a reliable muscle synergy extraction, the following steps were taken to establish the minimal number of trials in each task before averaging. Firstly, the similarities of synergy structures between any two trials for each task were estimated by Pearson's correlation coefficients (r). For the *i*-th trial, there were S-1 correlation coefficients expressed as R_*i*_ = [r_1_, r_2_,…,r_S−1_], and the average of R_*i*_ was calculated to represent the level of synergy structures similarity between the *i*-th trial and other trials. Then the dispersion of all the averaged R_*i*_ was analyzed using the quartile method (Q1:1st quartile, Q2:2st quartile, Q3:3st quartile), and trials corresponding to the outliers lower than Q1−1.5^*^(Q3−Q1) were removed. Finally, muscle synergies were averaged across the remained trials.

#### Quantitative similarity of muscle synergies

Similarity between two muscle synergies matrices or two activation coefficient matrices was determined by correlation coefficient r. For two synergy matrices ***W****1* = [*w1*_1_,*w1*_2_,*…,w1*_*n*1_] and ***W****2* = [*w2*_1_,* w2*_2_,*…,w2*_*n*2_] (*n1* and *n2* represent the synergy number, n1≤n2), synergy structure similarity coefficient *r*_*W*−2_ was defined as formula (3). For two activation coefficient matrices ***C****1* = [*c1*_1_,*c1*_2_,*…,c1*_*n*1_] and ***C****2* = [*c2*_1_,*c2*_2_,*…,c2*_*n*2_], activation pattern similarity coefficient *r*_*C*−2_ was defined as formula (4). Considering ***W*** and ***C*** simultaneously, *r*_*task*−2_ was defined as formula (5) to represent the similarity between two tasks. Based on the definition, synergy-related parameters (*r*_*W*−2_, *r*_*C*−2_, and *r*_*task*−2_) all range from 0 to 1, and large value means high similarity.

(3) $$\begin{array}{r} r_{W\text{-}2}\left(W1,W2\right)\text{=}\frac{1}{n_{1}}\overset{n_{1}}{\underset{i=1}{\sum }}max\left[r\left(w1_{i},w2_{j}\right){|}_{j=1}^{n_{2}}\right] \end{array}$$

(4) $$\begin{array}{r} r_{C\text{-}2}\left(C1,C2\right)\text{=}\frac{1}{n_{1}}\overset{n_{1}}{\underset{i=1}{\sum }}max\left[r\left(c1_{i},c2_{j}\right){|}_{j=1}^{n_{2}}\right] \end{array}$$

(5) $$\begin{array}{r} r_{task\text{-}2}=\left(r_{W\text{-}2}+r_{C\text{-}2}\right)/2 \end{array}$$

Similarly, synergy structure similarity coefficient *r*_*W*−3_ was defined as formula (6) for three synergy matrices, and activation pattern similarity coefficient *r*_*C*−3_ was defined as formula (7) for three activation coefficient matrices. Considering ***W*** and ***C*** simultaneously, *r*_*task*−3_ was defined as formula (8) to represent the similarity between three tasks. Here **T** represents the number of task which is equal to 3. Based on the definition, *r*_*W*−3_, *r*_*C*−3_ and *r*_*task*−3_ all range from 0 to 1, and large value means high similarity.

(6) $$\begin{array}{r} r_{W\text{-}3}\text{=}\overset{T-1}{\underset{i=1}{\sum }}\overset{T}{\underset{j=i+1}{\sum }}\left[r_{W\text{\_}2}\left(W1_{i},W2_{j}\right)\right]/T \end{array}$$

(7) $$\begin{array}{r} r_{C\text{-}3}\text{=}\overset{T-1}{\underset{i=1}{\sum }}\overset{T}{\underset{j=i+1}{\sum }}\left[r_{C\text{\_}2}\left(C1_{i},C2_{j}\right)\right]/T \end{array}$$

(8) $$\begin{array}{r} r_{task\text{-}3}=\left(r_{W\text{\_}3}\text{+}r_{C\text{\_}3}\right)/2 \end{array}$$

#### Upper limb assessment metrics

In this study, muscle synergy analysis of three upper limb motion tasks was conducted on TD group and CP group. *r*_*W*−2_, *r*_*C*−2_, and *r*_*task*−2_ were defined to reflect the differences in number, structure and activation pattern of the extracted muscle synergies between CP group and TD group. In the following data analysis, *r*_*W*−2_ was found to have high ability to depict the inter-subject similarity within task and the intra-subject similarity between tasks. Metrics based on either *r*_*W*−2_ combination of a single task, or the average of *r*_*W*−2_ across any two tasks, or the average of *r*_*W*−2_ across all 3 tasks were to assess the upper limb motor dysfunction of CP children. Thus, there were seven (${\text{C}}_{3}^{1}$ + ${\text{C}}_{3}^{2}$ + ${\text{C}}_{3}^{3}$) possible metrics (Table 2).

**Table 2:** **The upper limb assessment (***UPA***) metrics**.

**UPA**	***UPA*(1)**	***UPA*(2)**	***UPA*(3)**	***UPA*(4)**	***UPA*(5)**	***UPA*(6)**	***UPA*(7)**
Expressions	*r*_*W*−2_ (1)	*r*_*W*−2_ (2)	*r*_*W*−2_ (3)	(*r*_*W*−2_ (1)+ *r*_*W*−2_ (2))/2	(*r*_*W*−2_ (2)+ *r*_*W*−2_ (3))/2	(*r*_*W*−2_ (1)+ *r*_*W*−2_ (3))/2	(*r*_*W*−2_ (1)+ *r*_*W*−2_ (2)+ *r*_*W*−2_ (3))/3

#### Statistics methods

In this study, descriptive statistics included the calculation of the mean and standard deviation. Independent sample *T*-test was used to analyze whether there existed difference in VAF, synergy structure and activation coefficients between the CP group and the TD group. One-way ANOVA was used to evaluate the inter-group and inter-group differences of *r*_*W*−2_, *r*_*C*−2_, and *r*_*task*−2_ coefficients. Reported results were considered significant for *p* < 0.05.

## Results

### Muscle synergy analysis of healthy subjects

**Figure 3:**
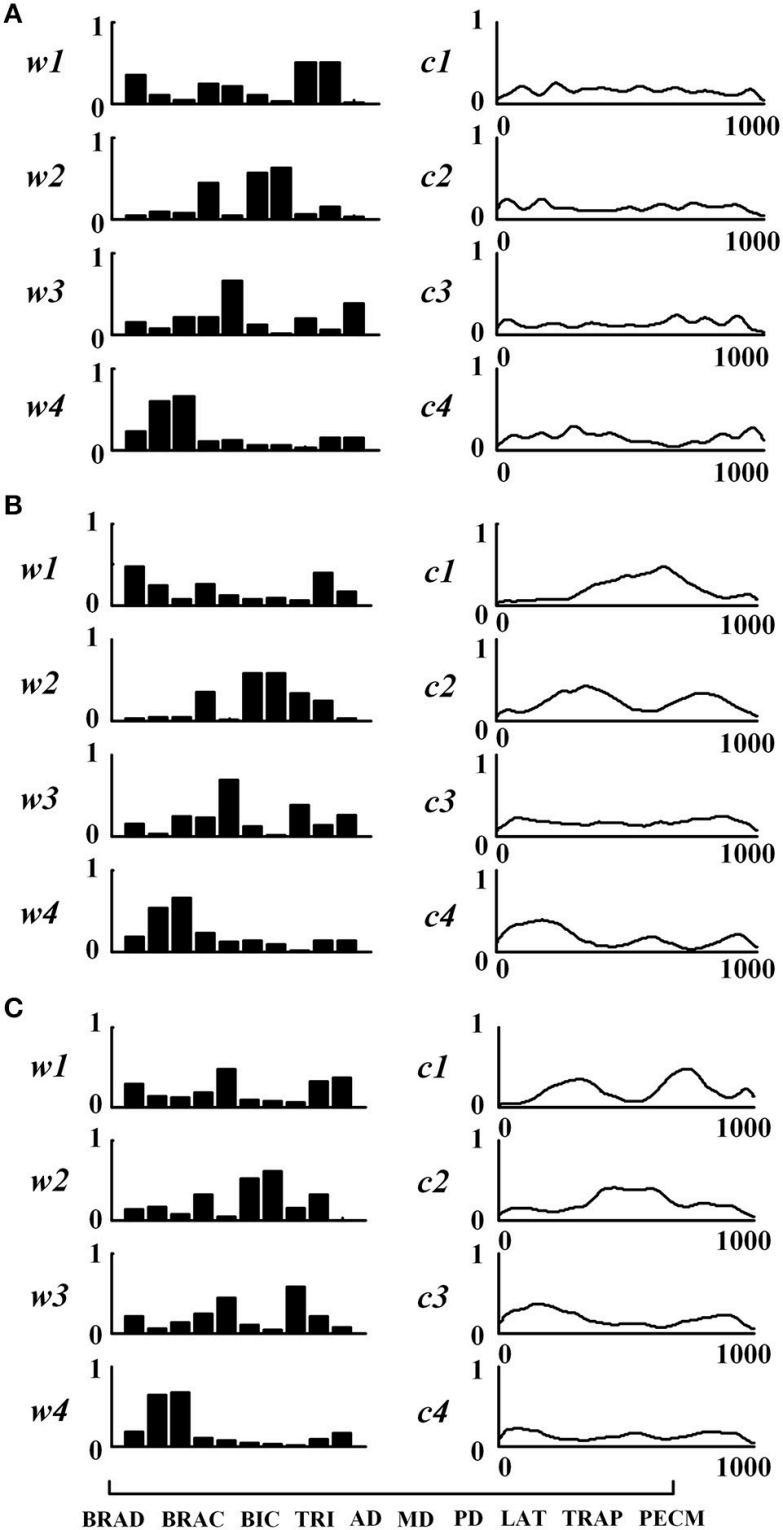
**Muscle synergies extracted from subject TD1. (A)** Task 1, **(B)** Task 2, and **(C)** Task 3. For each task, muscle synergies (w_1_ ~w_4_) and their corresponding activation patterns (c_1_ ~c_4_) are given. For muscle synergies, the horizontal axis corresponds to 10 selected muscles as listed in the lower-left of the figure.

Figure 3 shows the extracted muscle synergies and the corresponding activation patterns from three tasks of one healthy subject. Four muscle synergies were recruited in three tasks. In Task 1, the first synergy (w1) mainly reflects the activity of BRAD, LAT, and TRAP; the second synergy (w2) consists of TRI, MD, and PD; the third synergy (w3) is mainly characterized by AD and PECM whereas the fourth synergy (w4) is loaded by BRAC and BIC. Similarly, the other two tasks also recruited four muscle synergies. Examining Figure 3 carefully, high structure similarities can be found between the three similar tasks (*r*_*W*−3_ = 0.92), however, the activation patterns are different (*r*_*C*−3_ = −0.08).

**Figure 4:**
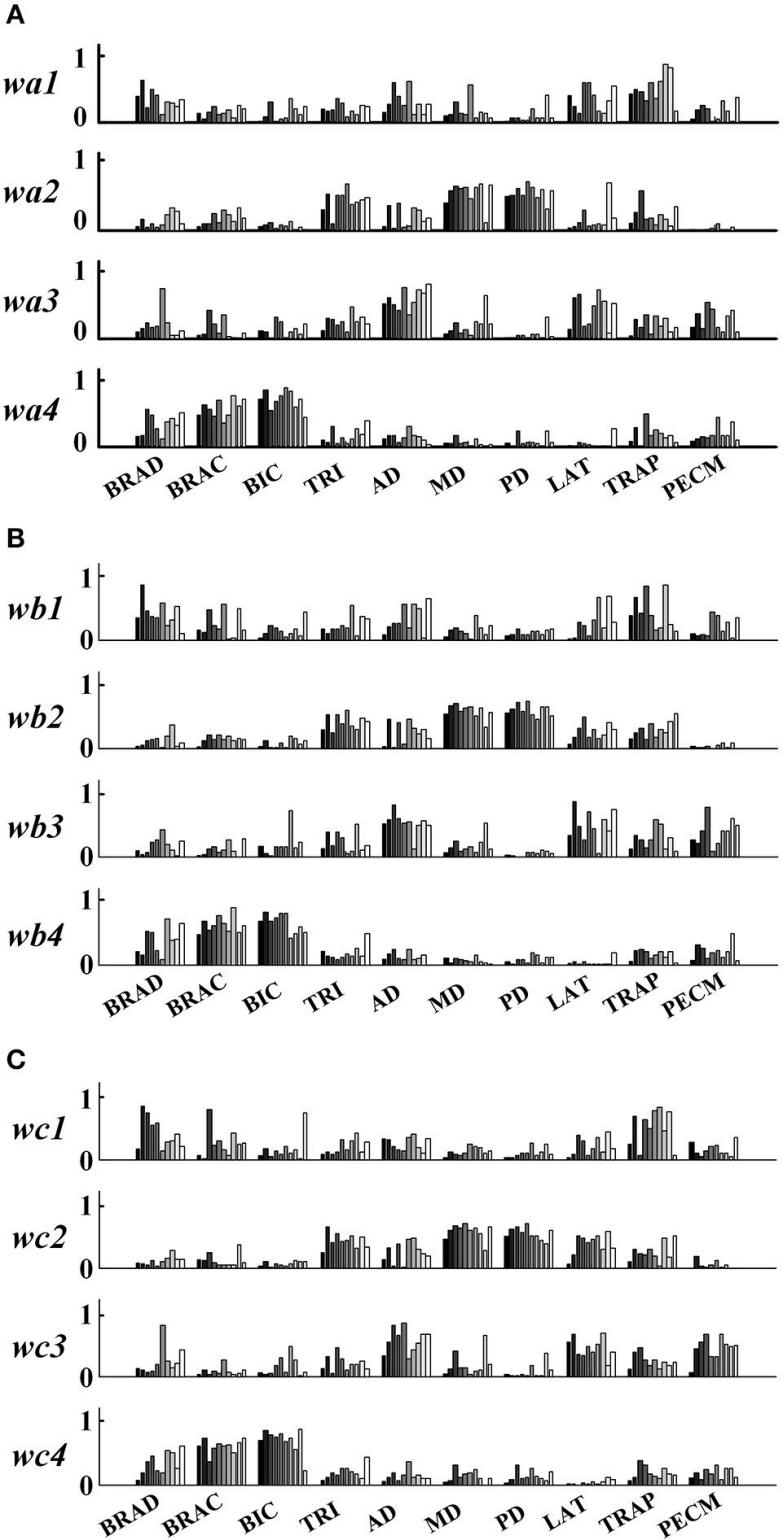
**Muscle synergies extracted from 10 healthy children. (A)** Task 1, **(B)** Task 2, and **(C)** Task 3. Ten groups in the horizontal axis corresponds to 10 muscles, and each group contains data from 10 subjects (left to right: TD 1~TD 10).

**Figure 5:**
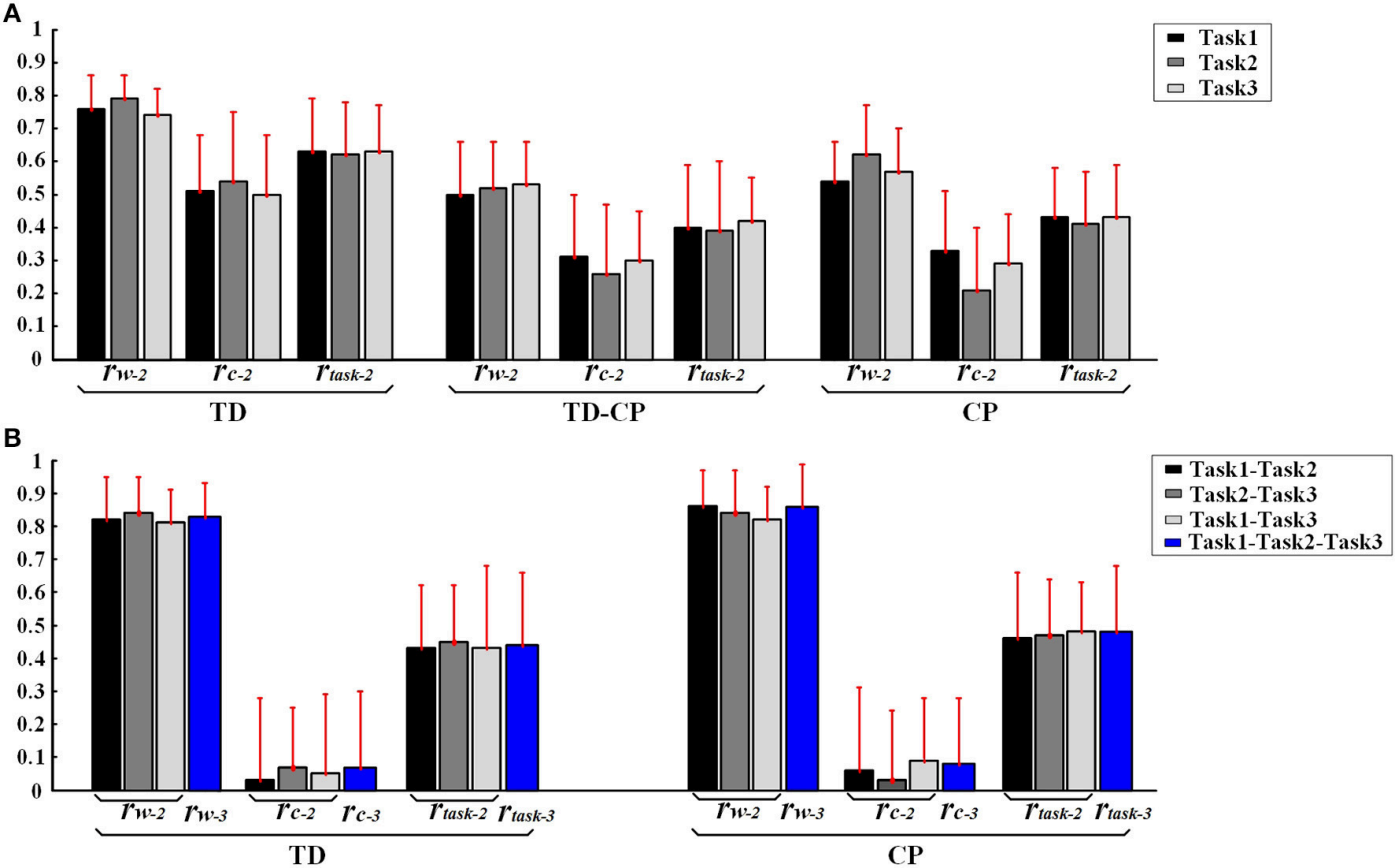
**(A)** Inter-subject similarity coefficients (*r*_*W*−2_, *r*_*C*−2_, *r*_*task*−2_.) in three task. **(B)** Intra-subject similarity coefficients between two tasks and Intra-subject similarity coefficients between three tasks of TD and CP groups. Error bars represent the standard deviation of synergy-related parameters.

In order to further explore the structure characteristics of muscle synergies between subjects, the similar muscle synergies in each task were grouped based on the maximum value of *r*. In this process, muscle synergy of the first subject (TD1) was selected as the template, and the muscle synergies from the remaining subjects were grouped based on the best-matching of ***W*** matrix. After grouping, although there were differences between subjects, certain regularity in each synergy could be found in a macroscopic scale as shown in Figure 4. Take the muscle synergy extracted in Task 1 as example (Figure 4A), the first synergy (*w*_*a*1_) mainly reflects the activity of LAT and TRAP; the second synergy (*w*_*a*2_) reflects the activity of TRI, DELA, DELM, and DELP; the third synergy (*w*_*a*3_) consists of DELA and PECM whereas the fourth synergy (*w*_*a*4_) consists of BRAD, BRAC, and BIC. For Task 2 and Task 3, the same macroscopic scale signified obvious regularity after grouping the similar structure of muscle synergies. As shown in Figure 5A, the inter-subject structure similarity coefficient *r*_*W*−2_ is 0.76 ± 0.10 for Task 1, 0.79 ± 0.07 for Task 2, and 0.74 ± 0.08 for Task 3 in TD group. It demonstrates that the structure of the muscle synergies extracted from different TD subject in the same task has high similarity. However, less similarity exists in the activation patterns, and the inter-subject activation pattern similarity coefficient r_C−2_ is 0.51 ± 0.17 for Task 1, 0.54 ± 0.21 for Task 2, and 0.50 ± 0.18 for Task 3.

Furthermore, high muscle synergy structure similarity was observed between three similar tasks in TD group. As shown in Figure 5B, the intra-subject synergy structure similarity coefficient *r*_*W*−2_ is 0.82 ± 0.13 between Task 1 and Task 2, 0.84 ± 0.11 between Task 2 and Task 3, and 0.81 ± 0.10 for Task 1 and Task 3 in TD group, with the three-task synergy structure similarity coefficient r_W−3_ of 0.83 ± 0.10. However, no similarity can be found in the synergy activation patterns based on the intra-subject synergy activation pattern coefficients r_C−2_ and r_C−3_.

### Muscle synergy analysis of CP group

**Figure 6:**
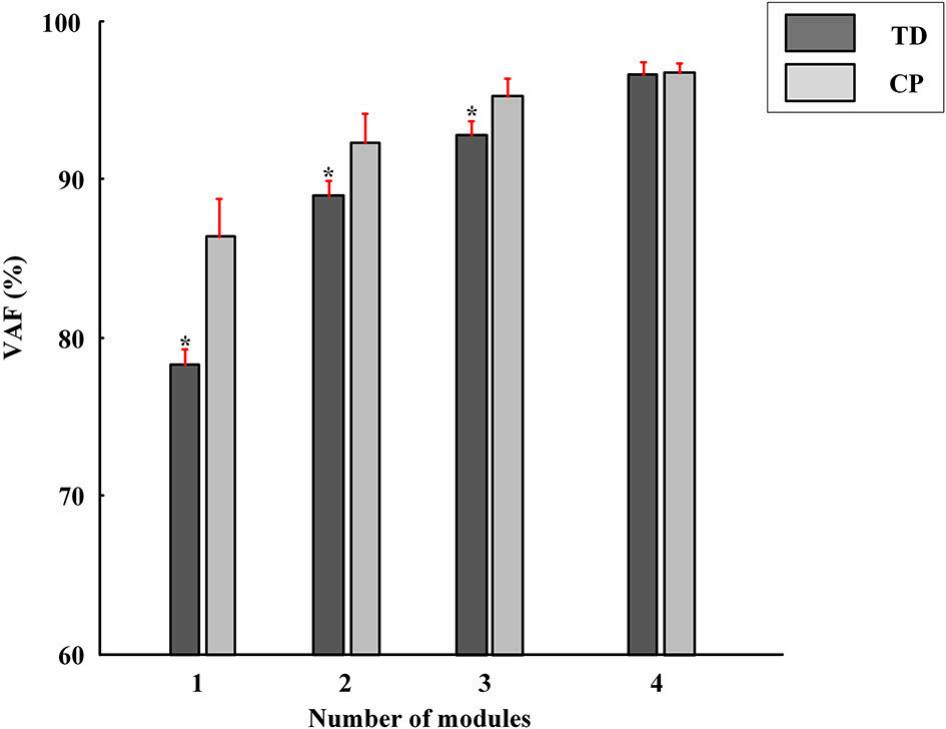
**The VAF corresponding to different number of muscle synergies (red bars, Mean ± SD)**. The VAF of TD group is significantly lower than that of CP group when the number of the extracted muscle synergies were <4 (^*^P < 0.01, Independent samples *T*-test).

**Figure 7:**
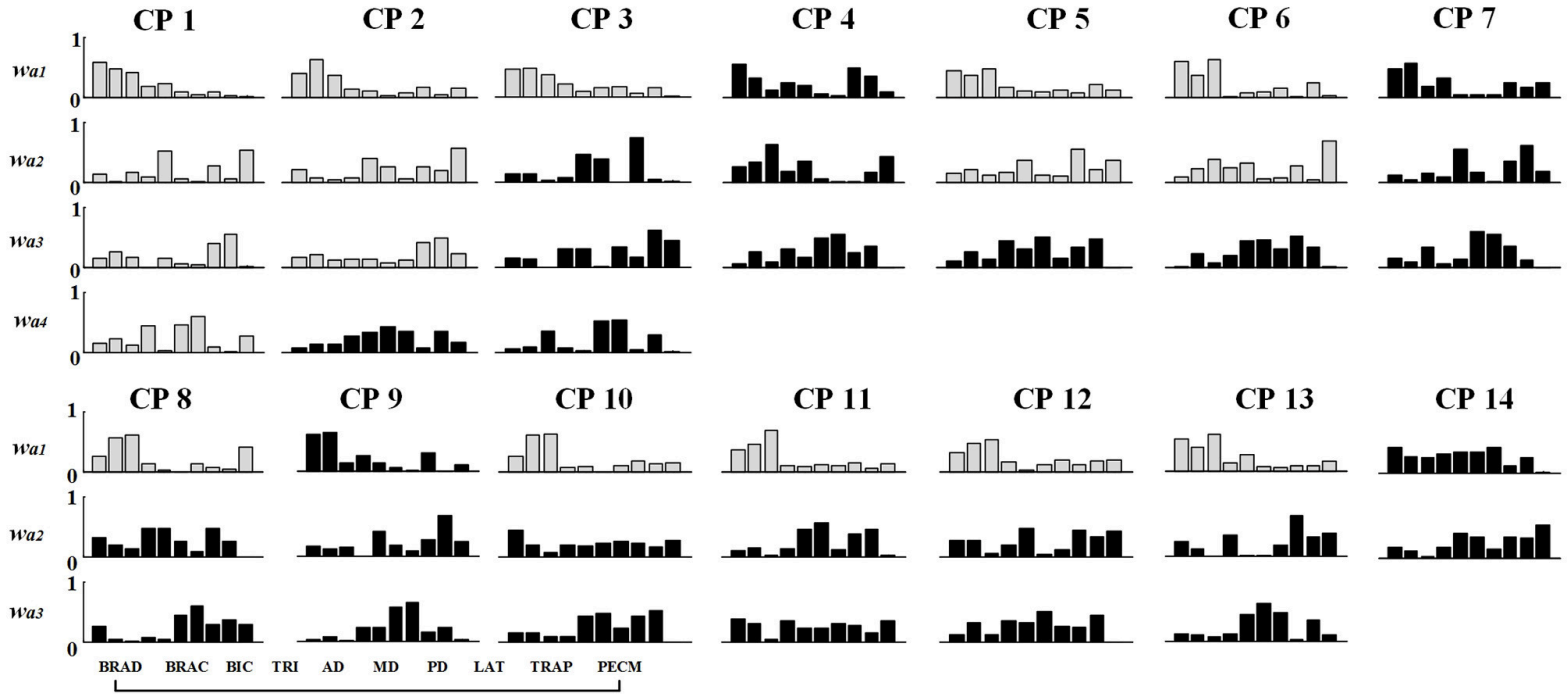
**Muscle synergy structures extracted from Task 1 in CP group**. New muscle synergies appeared in CP group were marked in black and the muscle synergies similar to TD group (*r* > 0.80) were marked in gray.

Differing from the TD group, CP children recruit 2~4 muscle synergies in each task (Table 3). As shown in Figure 6, the VAF of control group is significantly lower than that of CP group when the number of the extracted muscle synergies were <4 (*p* < 0.01, Independent sample *T*-test). The extracted muscle synergies of CP group in Task1 were shown in Figure 7. Three subjects (CP1~CP3) recruited four synergies, ten subjects (CP4~CP13) recruited three synergies and one subject (CP14) recruited two synergies. Subjects CP4~CP7 and CP10~CP13 recruited three synergy in each task (Table 3). As shown in Figure 5A, the inter-subject structure similarity coefficient *r*_*W*−2_ is 0.54 ± 0.12 for Task 1, 0.62 ± 0.15 for Task 2, and 0.57 ± 0.20 for Task 3 in CP group. It demonstrates that the structure of the muscle synergies extracted from CP subjects in the same task has moderate degree of similarity. Moreover, very low similarity exists in the activation patterns because the inter-subject activation pattern similarity coefficient r_C−2_ is 0.33 ± 0.18 for Task 1, 0.21 ± 0.19 for Task 2, and 0.29 ± 0.15 for Task 3, respectively. For CP group, high muscle synergy structure similarity was observed between the three similar tasks. As shown in Figure 5B, the intra-subject two synergy structure similarity coefficient *r*_*W*−2_ is 0.86 ± 0.11 between Task 1 and Task2, 0.84 ± 0.13 between Task 2 and Task 3, and 0.82 ± 0.10 between Task 1 and Task 3, along with the three synergy structure similarity coefficient r_W−3_ of 0.83 ± 0.10 in CP group. However, no similarity can be found in the synergy activation patterns based on the intra-subject synergy activation pattern coefficients r_C−2_ and r_C−3_.

**Table 3:** **Number of the extracted muscle synergies in CP group**.

	**CP1**	**CP2**	**CP3**	**CP4**	**CP5**	**CP6**	**CP7**	**CP8**	**CP9**	**CP10**	**CP11**	**CP12**	**CP13**	**CP14**
Task1	4(4)	4(3)	4(1)	3 (0)	3 (2)	3 (2)	3 (0)	3 (1)	3 (0)	3 (1)	3 (1)	3 (1)	3 (1)	2 (0)
Task2	4(4)	4(3)	4(0)	3 (0)	3 (1)	3 (2)	3 (0)	4 (1)	4 (3)	3 (1)	3 (1)	3 (0)	3 (1)	2 (0)
Task3	4(4)	4(3)	4(0)	3 (0)	3 (1)	3 (2)	3 (0)	4 (1)	3 (1)	3 (1)	3 (1)	3 (1)	3 (1)	2 (0)

^*^The number in brackets represents the number of synergies similar to TD group.

### Synergy similarity comparison between TD group and CP group

As shown in Table 3 and Figure 7, although some CP subjects could recruit muscle synergies similar to TD group, new muscle synergies with various structures appeared in CP group. In Task 1, subjects CP5~6 recruited two synergies that were also observed in TD group, but CP3, CP8, and CP10~13 only recruited one. In Task 2, subject CP6 recruited two synergies that were also observed in TD group, but CP5, CP8, CP10, CP13 only recruited one. In Task 3, subjects CP6 recruited two synergies that were also observed in TD group, but CP8 and CP10~13 only recruit one. CP1 and CP2 recruited four or three muscle synergies similar to TD group in three tasks, and other CP subjects including CP4, CP7, and CP14 recruited no synergies similar to TD group. As shown in Figure 5A, the TD-CP structure similarity coefficient *r*_*W*−2_ is 0.50 ± 0.16 for Task 1, 0.52 ± 0.14 for Task 2, and 0.53 ± 0.13 for Task 3. It demonstrates that the structure of the muscle synergies extracted from TD group and CP group in the same task has moderate degree of similarity. Moreover, very low similarity exists in the activation patterns because the TD-CP activation pattern similarity coefficient *r*_*C*−2_ is 0.31 ± 0.19 for Task 1, 0.26 ± 0.21 for Task 2, and 0.30 ± 0.15 for Task 3, respectively.

In Figure 5A, TD group has higher inter-subject structure similarity coefficient *r*_*W*−2_ than TD-CP and CP, so do the activation pattern similarity coefficient r_C−2_ and two task synergy similarity coefficient r_task−2_ (*p* < 0.05, One way ANOVA). These results show obvious synergy structure and activation pattern differences between TD group and CP group, and individual differences in CP group are larger than that of TD group. For intra-subject similarity coefficients r_W−3_, r_C−3_, and r_task−3_ show no significant difference between CP group and TD group (*p* > 0.05, Independent sample *T*-test). Based on above results, three synergy-related parameters *r*_*W*−2_, r_C−2_, and r_task−2_ can reflect the difference between TD group and CP group.

### Assessment of CP upper limb motor function

**Figure 8:**
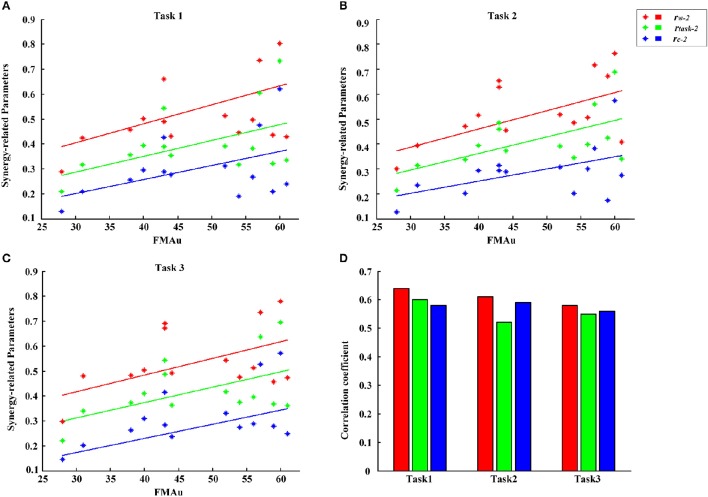
**FMAu scores vs. the synergy-related parameters for 14 CP subjects**. **(A)** Task 1, **(B)** Task 2, **(C)** Task 3. Different colors of points represent data for one synergy-related parameters. Different colors of lines represent linear fitting curve. **(D)** Pearson's correlation coefficients were calculated between three synergy-related parameters and FMAu scores.

According to the differences in the number, structure and activation pattern of the extracted muscle synergies between CP group and TD group in three tasks, we tried to explore a feasible parameter to assess the upper limb motor dysfunction of CP children. For each task, synergy-related parameters between each CP subject and ten typically developed children were calculated firstly, and then 10 values of *r*_*W*−2_, r_C−2_, and r_task−2_ were averaged, respectively, to represent the three synergy-related parameters of each CP subject. Figure 8 shows the relationship between three synergy-related parameters and FMAu scores for 14 CP subjects. In detail, CP1 with the largest synergy-related parameters (*r*_*W*−2_ = 0.81, r_C−2_ = 0.60, r_task−2_ = 0.71) got high FMAu score (60) and CP14 with the lowest synergy-related parameters (*r*_*W*−2_ = 0.29, r_C−2_ = 0.11, r_task−2_ = 0.21) got the smallest FMAu score (28) in Task 1. Other two tasks also presented the same results. Only moderate degree of relevance were found between three synergy-related parameters and FMAu scores (0.55<*r*<0.64, Figure 8D) as five CP subjects (CP4, CP6, CP7, CP9, CP12) got obviously high FMAu scores but low synergy-related parameters in three tasks.

**Figure 9:**
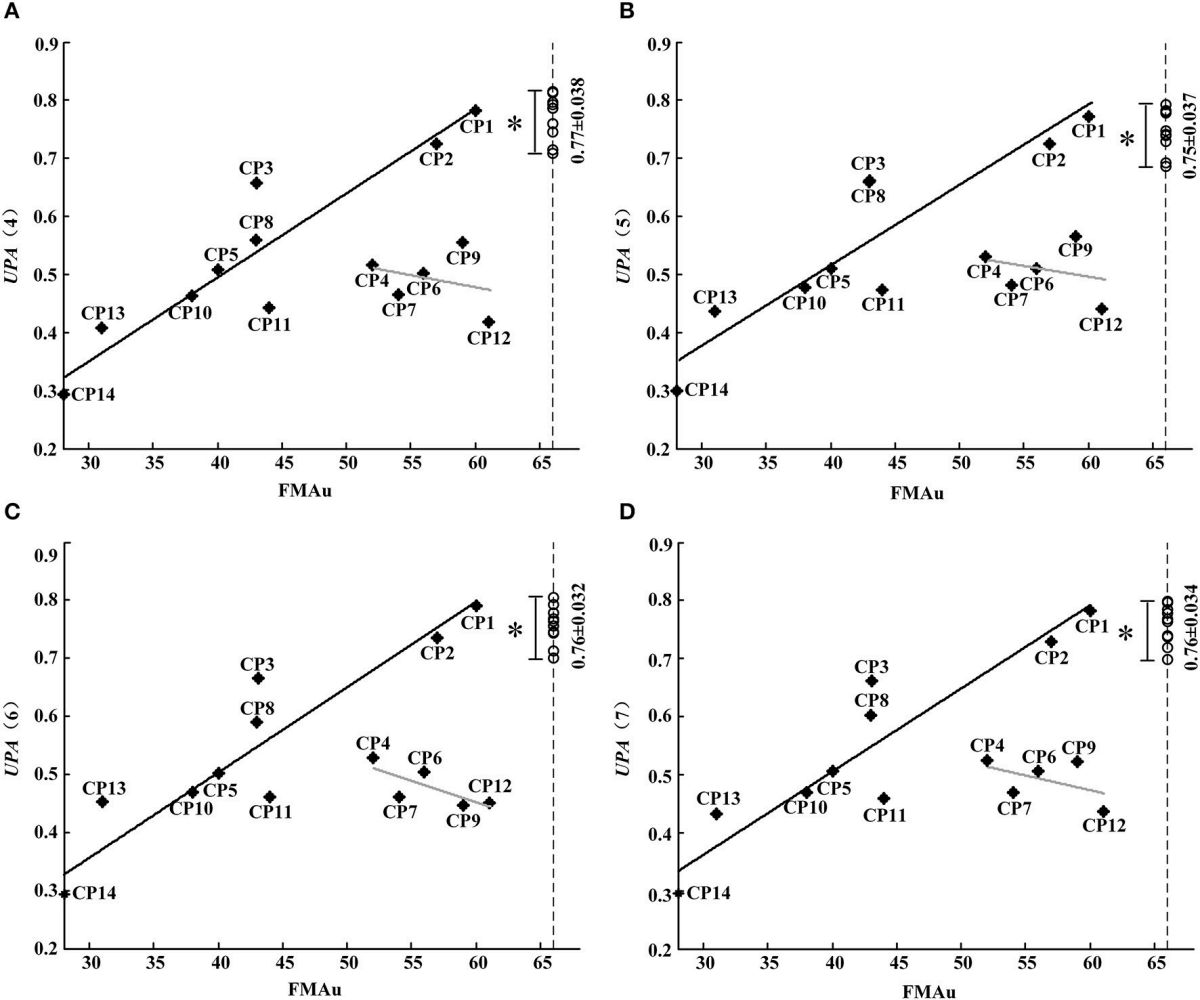
**FMAu scores vs. the UPAs cores. (A)** UPA (4), **(B)** UPA(5), **(C)** UPA (6), **(D)** UPA(7). Point represents CP subject and circle represents TD subject (UPA score of TD group: Mean ± SD). Black lines are the linear fitting curves of eight subjects and gray lines are the curves of other subjects. UPA scores of TD group are significantly higher than that of CP group (^*^P < 0.05, Independent samples *T*-test).

Although synergy-related parameters presented moderate correlation with FMAu scores, three synergy-related parameters could reflect the upper limb motor function of CP children to some extent. As shown in Figure 5, *r*_*W*−2_ has high ability to depict the inter-subject similarity within task and the intra-subject similarity between tasks. Therefore, *UPA* metrics under different combinations of *r*_*W*−2_ of three tasks (Table 2) were defined to quantitatively assess muscle synergies abnormality in this study. Using the *UPA* metrics, CP children obtained significantly lower scores than TD children in each task (*p* < 0.05, Independent sample *T*-test). As shown in Figure 9, the *UPA*(4)~*UPA*(7) scores of eight subjects (CP1, CP2, CP3, CP5, CP8, CP10, CP11, and CP14) are positively related to FMAu, but five subjects (CP4, CP6, CP7, CP9, and CP12) show weak relation between the *UPA* scores and FMAu scores. Furthermore, there is no significant difference among parameters *UPA*(1)~*UPA*(7) for the assessment of upper limb motor dysfunction (*p* < 0.05, One way ANOVA).

## Discussion

Based on muscle synergy analysis of three upper limb motion tasks in TD group and CP group, the main contribution of this study is to propose a quantitative assessment method for upper limb motor dysfunction of CP children. The research results verified that muscle synergy analysis has a great potential in the assessment of motor impairment.

### Muscle synergy differences between TD group and CP group

For each subject in TD group, four muscle synergies were extracted in each task, and high structure similarities with different activation patterns existed between three similar tasks. According to muscle synergy hypothesis, muscle synergies represent a library of motor subtasks, which can be combined flexibly by the nervous system to produce complex and natural movements. Muscle synergy analysis results obtained in this study provided new evidence to support the hypothesis that diverse motor behaviors were generated by recruiting certain muscle synergies in different activation ways. For TD group, four muscle synergies extracted from three tasks represent part of the library of motor tasks regarding the flexing and extending movements of elbow and shoulder joints. Compared to the TD group, large muscle synergy differences appeared in CP group, in term of reduced synergy number, altered synergy structures and activation patterns. Previous studies reported that CP children recruited fewer synergies during gait than typically developing children (Schwartz et al., 2014; Tang et al., 2014), suggesting that individuals with CP used a simpler neuromuscular control strategy. From the aspect of upper limb movements, the results of this study support the opinion that individuals with CP might be used simpler neuromuscular control strategy to finish related motion tasks.

### Reliability of CP upper limb motor dysfunction assessment

Since changes of muscle synergy in number and structure can be used to examine various pathological changes in the CNS, muscle synergy analysis has been suggested as a metric for motor assessment (Safavynia et al., 2011; Schwartz et al., 2016). In this study, the difference of muscle synergies in number, structure, and activation pattern were found to be related generally to clinical FMAu. CP1 with the highest FMAu score (FMAu = 60) recruited four muscle synergies in all three tasks, CP5 with a moderate FMAu score (FMAu = 40) recruited three synergies, and CP14 with the smallest FMAu score (FMAu = 28) only recruited two synergies in all three tasks. The clinical manifestation of CP1 is spastic paralysis of the lower limb. Her upper limb motor function is much better than lower limb (FMAu = 60). During the experiment in this study, CP1 presented low functional impairment level and completed three tasks smoothly. In other words, CP1 could complete the task in a controlled manner. CP5 has continuous muscle tension of upper limb, his main clinical manifestations include moderate flexion of the elbow and wrist. The synergy number of CP5 is <TD subjects and CP1. CP14 has the worst motor function, and he was diagnosed with spastic quadriplegia and classified as Grade III with the lowest FMAu (28). His clinical manifestations include slight adduction and internal rotation of the shoulder, severe flexion of the elbow and wrist, flexion of the fingers, and adduction of the thumb. Due to the synergic movements caused by no separation of multi-joint movements, only two muscle synergies were recruited. Moreover, considering the recruit ability of CP subjects, the number of recruited muscle synergies that is similar to TD group might also reflect the degree of the upper limb dysfunction. For instance, CP1 recruited the same number and similar structure of synergies to healthy subject in the three tasks. CP 14 recruited two muscle synergies in each task, and none of these synergies were observed in TD group.

Considering the combinations of three tasks, seven *UPA* metrics defined based on *r*_*W*−2_ were proposed to identify motor dysfunction in this study. CP children obtained significantly lower *UPA* scores than TD children, and the assessment scores of *UPA* and FMAu were positively related in eight CP subjects. This result demonstrated that *UPA* metrics could be used effectively to evaluate upper limb motor dysfunction of CP subjects. On the other hand, FAMu scales consists of reflex testing, movement observation, grasping testing and coordination assessment of the function of hand and independent joints such as shoulder, elbow, forearm, and wrist (Davids et al., 2006). However, muscle synergy analysis pays more focus on the coordination assessment of joints in special motion tasks. The proposed *UPA* metrics were found to be more effective than FMAu in measuring arm motor dysfunction caused by the muscular rigidity, quiver in limbs and tilt in head in CP children based on the following results. (1) Subjects CP4, CP6, CP7, and CP12 were with spastic and their thumbs existed various degrees of adduction and muscular rigidity. During the motion task, these four subjects could not complete task very smoothly due to the combined joint abnormal activity caused by muscular rigidity, and obtained very low *UPA* assessment scores (CP4: *UPA*_mean_ = 0.52; CP6: *UPA*_mean_ = 0.51; CP7: *UPA*_mean_ = 0.47; CP12: *UPA*_mean_ = 0.35). However, they obtained relatively high FMAu scores (CP4: 52; CP6: 56; CP7:54; CP12:61). This result meant bad hand/wrist motor function and muscular rigidity of these CP subjects could not be measured comprehensively in FMAu scale. (2) The main clinical manifestation of CP9 was quiver in limbs and head, which leaded to the poor stability of motion. Due to quiver usually could not affect the flexing/extending activity of individual joint, CP9 obtained high score with FMAu assessment (FMAu = 59). However, when assessed with *UPA* metrics, CP9 got low scores (*UPA*_mean_ = 0.52). It demonstrated that the impact of quiver on muscles activities could be reflected in UPA metrics. (3) CP12 was with the same CP type and GMFCS level as CP1. CP12's head tilted severely to one side and such manifestation leaded to abnormal movements of his shoulder joint. CP12 and CP1 obtained similar FMAu scores (CP12: 61, CP1:60) but CP12 obtained lower *UPA* scores (*UPA*_mean_ = 0.44) than CP1 (*UPA*_mean_ = 0.78). This result demonstrated that *UPA* metrics were superior to FMAu in measuring the abnormal movements of shoulder joint.

By now, the standardized assessment scales for motor function measuring mainly relied on clinicians' own visual sense or the self-report of patient (Foley et al., 2003). Different clinicians would possibly give different scores for the same patient, and the outcome would be vague and inaccurate. Some changes of the patient's motor function, such as neural mechanisms, might not be captured. Therefore, subjectivity and low-sensitivity were two main shortcomings of applying scales. Sometimes, such assessment scales may even be verbose and troubling. However, muscle synergy analysis based on sEMG data is a quantitative evaluation method for motor function. What we discussed above suggests that *UPA* metrics are both objective and convenient, and can provide more neuromuscular control information about arm motor dysfunction in CP children.

### Limitation and future work

This paper conducted a preliminary study work on the evaluation of upper limb motor dysfunction of CP children from the perspective of muscle synergy analysis. Although some interesting results have been obtained, there are some limitations waiting for further efforts. Firstly, only 10 typically developed children and 14 children with CP were involved in this study. As a small number of subjects may lead to restricted statistical findings, more subjects in different groups should be recruited in future study. Secondly, considering the small size of muscles of children, just 10 upper limb muscles were taken into account in the current study, which may hinder the understanding of the strategy of how the nervous system organizes movements of the whole body. More muscles should be considered in the future. Thirdly, three similar tasks defined in this study showed consistent assessment. Different tasks instead of similar tasks should be explored for the assessment of the motor function impairment of upper limb in patients with CP in the future. Finally, while muscle synergy analysis could provide more information about arm motor dysfunction in children with CP than FMAu scores, it might be a complicated method for clinical use.

## Conclusion

In this study, muscle synergy analysis of three upper limb motion tasks was conducted in typically developed children and CP children. TD group was found to recruit 4 muscle synergies in all three tasks. However, 2~4 mature synergies were recruited in CP group, and many abnormal structures specific to CP group appeared. In the three synergy-related parameters defined to depict the differences in structure and activation pattern of muscle synergies, structure similarity coefficient was verified to have high ability in depicting the inter-subject similarity within task and the intra-subject similarity between tasks. Seven *UPA* metrics, which were defined as the combinations of the structure similarity coefficients between the three tasks, were proposed and verified to be effective in assessing the upper limb motor function of CP children. The proposed assessment method can serve as a promising approach to derive a physiologically based quantitative index for upper limb motor function in CP clinical diagnosis and rehabilitation.

## Author contributions

LT and XC conceived and designed the experiments; LT and SC performed the experiments; LT analyzed the data; LT and XC wrote the paper; XC, GZ, and XZ supervised and reviewed the manuscript.

## Funding

This work was supported by the National Nature Science Foundation of China (NSFC) under Grant 61671417, 61431017, and 61271138.

### Conflict of interest statement

The authors declare that the research was conducted in the absence of any commercial or financial relationships that could be construed as a potential conflict of interest.
